# Signs of atopic dermatitis and contact dermatitis affected by distinct *H2*-haplotype in the NC/Nga genetic background

**DOI:** 10.1038/s41598-018-21049-x

**Published:** 2018-02-07

**Authors:** Kozo Ohkusu-Tsukada, Daiki Ito, Yuki Okuno, Teruyo Tsukada, Kimimasa Takahashi

**Affiliations:** 1 Department of Veterinary Pathology, Nippon Veterinary and Life-science University (NVLU), Musashino, Japan; 2 Radiation Biology Team, Nishina Center for Accelerator-based Science, RIKEN, Wako, Japan

## Abstract

We recently advocated in favour of naming a novel *H2-*haplotype consisting of K^d^, D/L^dm7^, I-A^k^ and I-E^k^ in the atopic dermatitis (AD) mouse model NC/Nga as “H-2^nc^.” The role of the *H2-*haplotype in AD development was investigated in *H2*^*b*^-congenic NC/Nga mice (NC.*h2*^*b/b*^ and NC.*h2*^*b/nc*^) established by backcrossing. A severe 2,4-dinitrofluorobenzene (DNFB)-induced dermatitis in NC/Nga was alleviated partially in NC.*h2*^*b/nc*^ and significantly in NC.*h2*^*b/b*^. The AD phenotype was correlated with thymic stromal lymphopoietin (TSLP)-epidermal expression levels and serum levels of total IgE and IL-18/IL-33. Histologically, allergic contact dermatitis (ACD) was accompanied by lymphocytes and plasma cells-infiltrating perivasculitis in NC.*h2*^*b/nc*^ and NC.*h2*^*b/b*^ and clearly differed from AD accompanied by neutrophils, eosinophils and macrophages-infiltrating diffuse suppurative dermatitis in NC/Nga. Interestingly, IFN-γ/IL-17 production from autoreactive CD4^+^ T-cells remarkably increased in DNFB-sensitised NC.*h2*^*b/b*^ but not in NC/Nga. Our findings suggest that AD or ACD may depend on haplotype H-2^nc^ or H-2^b^, respectively, in addition to the NC/Nga genetic background.

## Introduction

Atopic dermatitis (AD) is a common inflammatory skin disease caused by the interaction between genetic and environmental factors. NC*/*Nga mice are used as an AD-like disease model by many researchers because these mice spontaneously develop an AD-like disease under conventional condition, but not under specific pathogen-free (SPF) condition^1^. However, the possibility of inducing sustained dermatitis following application of 2,4-dinitrofluorobenzene (DNFB) in NC*/*Nga mice^2^ is important for research purposes using laboratory mice under SPF condition. The sustained application of DNFB on skin can elicit allergic contact dermatitis (ACD)-like disease (type IV allergy) through its role as a hapten^3^, and while BALB/c (H-2^d^), A/J (H-2^a^) and CBA mice (H-2^k^) are susceptible to DNFB-induced dermatitis development, C57BL/6 (H-2^b^), C57BL/10 (H-2^b^) and AB.Y mice (H-2^b^) are not, depending on the *H2*-haplotype^4^. Interestingly, DNFB-induced dermatitis in NC/Nga mice under SPF condition is associated with the development of AD-like disease (type I allergy) rather than ACD-like disease (type IV allergy)^2^. Because AD-like disease can develop in DNFB-applied BALB/c mice by adaptive transfer of immunoglobulin E (IgE) recognising DNFB as a hapten^5^, the immunoglobulin class switching to IgE is possible key for the development of AD-like disease. Interestingly, Interleukin (IL)-4 expression is not induced after repeated DNFB challenge in NC/Nga mice^2^. Even though IL-4 is a major cytokine for IgE-class switching^6^, the mechanism underlying the hyperproduction of IgE is not dependent on IL-4 levels in DNFB-treated NC/Nga mice but could rely on IL-13 inducing IL-4-independent IgE synthesis^7,8^. AD and ACD are both common skin diseases with an immune pathogenesis^9^ and histologically, include spongiotic lesions^10^. During clinical diagnosis, sometimes it is difficult to distinguish between AD and ACD because they present as eczematous dermatitis and may co-exist^10^. The relevance between AD and ACD has not been fully elucidated. Therefore, the DNFB-induced dermatitis model using NC/Nga mice and H-2^b^-congenic NC/Nga mice may represent an appropriate model for investigating the potential association between AD and ACD.

The genotype of AD sensitivity has been investigated in the NC/Nga genetic background. Genome-wide expression analysis between NC/Nga and BALB/c mice revealed the differential expression of more than 1,000 genes in the skin of mite antigen-exposed NC/Nga mice^11^. These included cytokines, cytokine receptors, proteases, and adhesion molecules. In humans, the deficiency of the skin barrier molecule filaggrin leads to alkalisation of the skin, favouring bacterial infections and increased metal ion–protein hapten complexes^12^. In NC/Nga mice having developed AD, the expression of filaggrin is low, but increases with treatment of an opioid analgesic drug JTC-801^13^. Our group recently found that the clonal deletion of T cell repertoires with specific T cell receptor Vβ chains is induced by the expression of two endogenous superantigens (*Mls-1*^*a*^*, MMTV(SHN)*) in the genetic background of NC/Nga mice^14^. This finding was in line with a previous report showing that under conventional condition, the Th1 dominant state mediated by IL-12 or IL-18 after *Staphylococcal enterotoxin B* (*SEB*) exposure was inhibited by the absence of Vβ8^+^ T-cells in NC/Nga mice, resulting in the induction of the Th2 dominant state^15^.

Genome-wide expression analysis indicates that besides inflammatory factors or the skin barrier system, the human genotype of AD sensitivity is also associated with MHC regions^16,17^. We recently proposed the name “H-2^nc^” for the novel *H2-*haplotype consisting of K^d^, D^dm7^/L^dm7^-hybrid mutant (D/L^dm7^), I-A^k^ and I-E^k^ in NC/Nga mice^18^. Here, H-2^b^ congenic NC/Nga mice (NC.*h2*^*b/b*^ or NC.*h2*^*b/nc*^) established by backcrossing (8 generations) were investigated for their sensitivity to DNFB-induced dermatitis. The role of the *H2-*haplotype in AD development in the NC/Nga strain is discussed.

## Results

### Establishment of H-2 congenic NC/Nga mice (NC/Nga)

**Figure 1:**
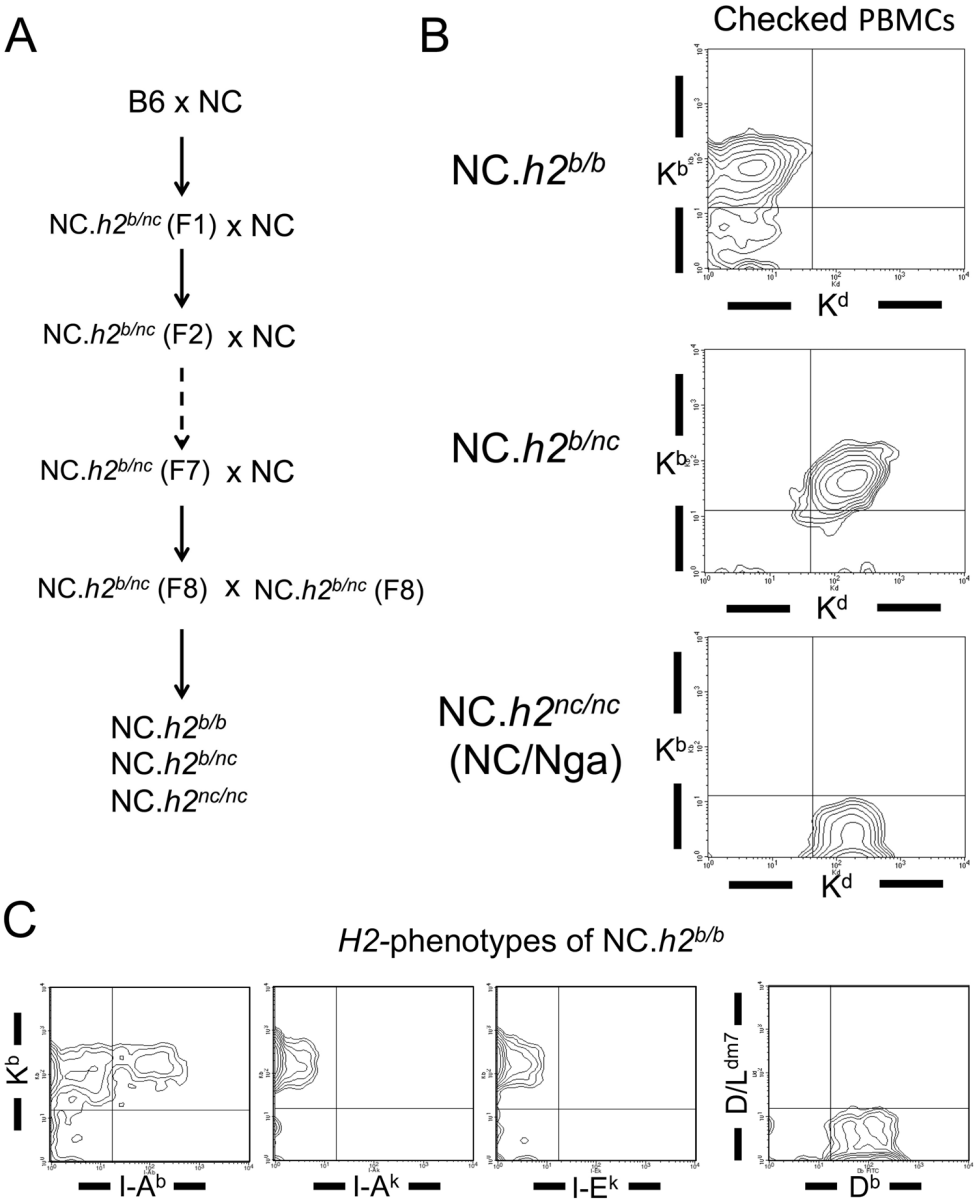
Establishment of H-2 congenic mice with NC/Nga background. **A**. The process of establishing H-2 congenic mice (NC.*h2*^*b/b*^, NC.*h2*^*b/nc*^ and NC.*h2*^*nc/nc*^) by NC/Nga backcross is indicated. **B**. H-2 congenic mice (NC.*h2*^*b/b*^, NC.*h2*^*b/nc*^ and NC.*h2*^*nc/nc*^) were sorted according to the expression pattern of K^d^ and K^b^ of PBMCs by flow cytometry. **C**. The *H2*-haplotype of NC.*h2*^*b/b*^ was checked based on the expression patterns of K^b^, D^b^, and I-A^b^ of PBMCs using flow cytometry.

To investigate whether the *H2*-haplotype (H-2^nc^) in NC/Nga mice (NC/Nga) is a cause for high sensitivity to AD-like disease, we established H-2 congenic mice (NC.*h2*^*b/b*^, NC.*h2*^*b/nc*^ and NC.*h2*^*nc/nc*^) by backcrossing (8 generations) the NC/Nga strain (Fig. 1A). Because the haplotype H-2^nc^ in NC/Nga strain shows the phenotype (K^d^, I-A^k^, I-E^k^)^18^, we tested peripheral blood mononuclear cells (PBMCs) from filial generation after backcrossing with NC/Nga strain by flow cytometry on the expression of K^d^ and K^b^. K^d^-negative and K^b^-positive populations, K^d^-positive and K^b^-positive populations and K^d^-positive and K^b^-negative populations in PBMCs indicate the haplotypes H-2^b^, H-2^b/nc^ and H-2^nc^, respectively (Fig. 1B). It was confirmed that the *H2*-phenotype of NC.*h2*^*b/b*^ shows K^b^, D^b^, and I-A^b^ (Fig. 1C).

### In DNFB-induced dermatitis model, the degrees of inflammation and dermatitis scores in NC.*h2*^*b/nc*^ and NC.*h2*^*b/b*^ are alleviated compared with that in NC/Nga

**Figure 2:**
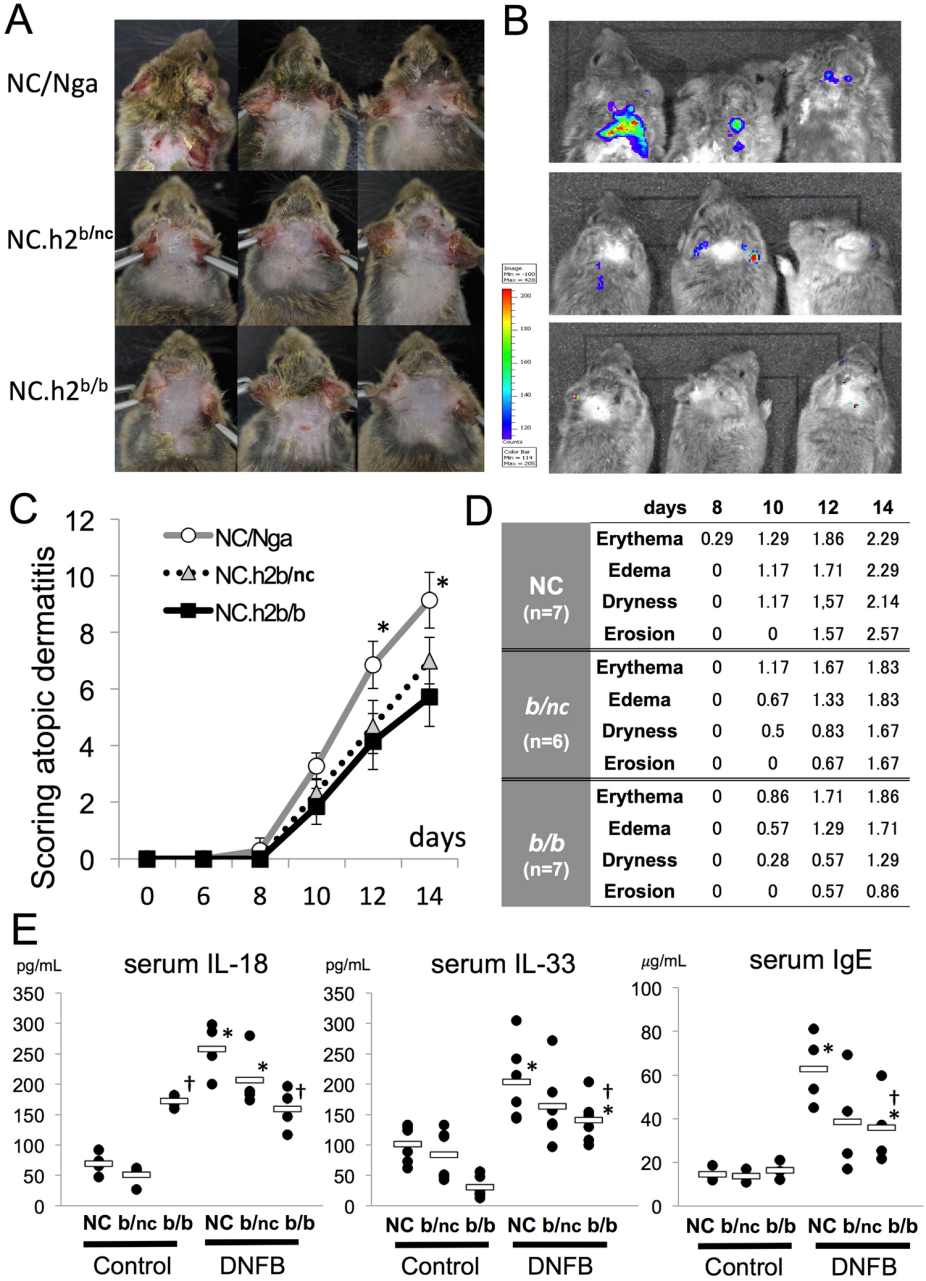
In DNFB-induced dermatitis, degrees of inflammation and dermatitis scores in NC.*h2*^*b/nc*^ and NC.*h2*^*b/b*^ are alleviated compared with NC/Nga. **A**. Gross lesion of DNFB-induced dermatitis shown for each H-2 congenic mice on day 14. **B**. MPO activity by *in vivo Imaging System* shown for each H-2 congenic mice on day 14. **C**. The gross lesion at day 14 of DNFB-induced dermatitis in each H-2 congenic mice was evaluated by a scoring index of atopic dermatitis. *NC/Nga *vs*. NC.*h2*^*b/b*^ or NC.*h2*^*b/nc*^, p < 0.05 *ANOVA*. **D**. The scoring index of DNFB-induced dermatitis in NC/Nga, NC.*h2*^*b/b*^ and NC.*h2*^*b/nc*^ are indicated. **E**. The concentration of serum IL-18, IL-33 and IgE in each mouse on day 14 were measured by ELISA method. *DNFB *vs*. control, p < 0.05, *ANOVA*. ^†^NC.*h2*^*b/b*^ *vs*. NC/Nga, p < 0.05, *ANOVA*.

DNFB-induced dermatitis in NC/Nga (or NC.*h2*^*nc/nc*^), NC.*h2*^*b/b*^ and NC.*h2*^*b/nc*^ was evaluated by both a scoring index of AD^19^ and the myeloperoxidase (MPO) activity using *in vivo Imaging System*. The degree of dermatitis score in DNFB-applied NC.*h2*^*b/b*^ was significantly alleviated compared with that in NC/Nga at days 12 and 14 (p < 0.05, *ANOVA*), whereas DNFB-applied NC.*h2*^*b/c*^ was partially alleviated (Fig. 2A,C). Particularly, dryness and erosion were strongly reduced in NC.*h2*^*b/b*^ and NC.*h2*^*b/c*^ mice compared with the effects seen in NC/Nga (Fig. 2D). The result of *in vivo Imaging System* at day 14 were in line with the degree of dermatitis scores, reflecting the degree of inflammation with MPO production due to the accumulation of activated neutrophils and macrophages (Fig. 2B).

### Serum IL-18, IL-33 and IgE produced by DNFB-induced dermatitis in NC.*h2*^*b/nc*^ and NC.*h2*^*b/b*^ are decreased compared with NC/Nga

Serum levels of IL-18 and IL-33 are generally known as mast cell activators, and when binding to Fc epsilon receptors (FcεRs) on the surface of mast cells, IgE stimulate antigen-specific degranulation. Serum levels of IL-18, IL-33 and IgE were significantly increased in NC/Nga, NC.*h2*^*b/nc*^ or NC.*h2*^*b/b*^ by DNFB-application against each control (p < 0.05, *ANOVA*). Serum levels of IL-18, IL-33 and IgE increased by DNFB-application were lower in NC.*h2*^*b/b*^ than in NC/Nga (p < 0.05, *ANOVA*) (Fig. 2E). Although there were no statistically significant differences, serum levels of IL-18 (p = 0.14, *ANOVA*), IL-33 (p = 0.16, *ANOVA*) and IgE (p = 0.064, *ANOVA*) in NC.*h2*^*b/c*^ tended to be lower than those in NC/Nga.

### Histologically, DNFB induces ACD-like lesions in NC.*h2*^*b/nc*^ and NC.*h2*^*b/b*^, whereas AD-like lesions are induced in NC/Nga

**Figure 3:**
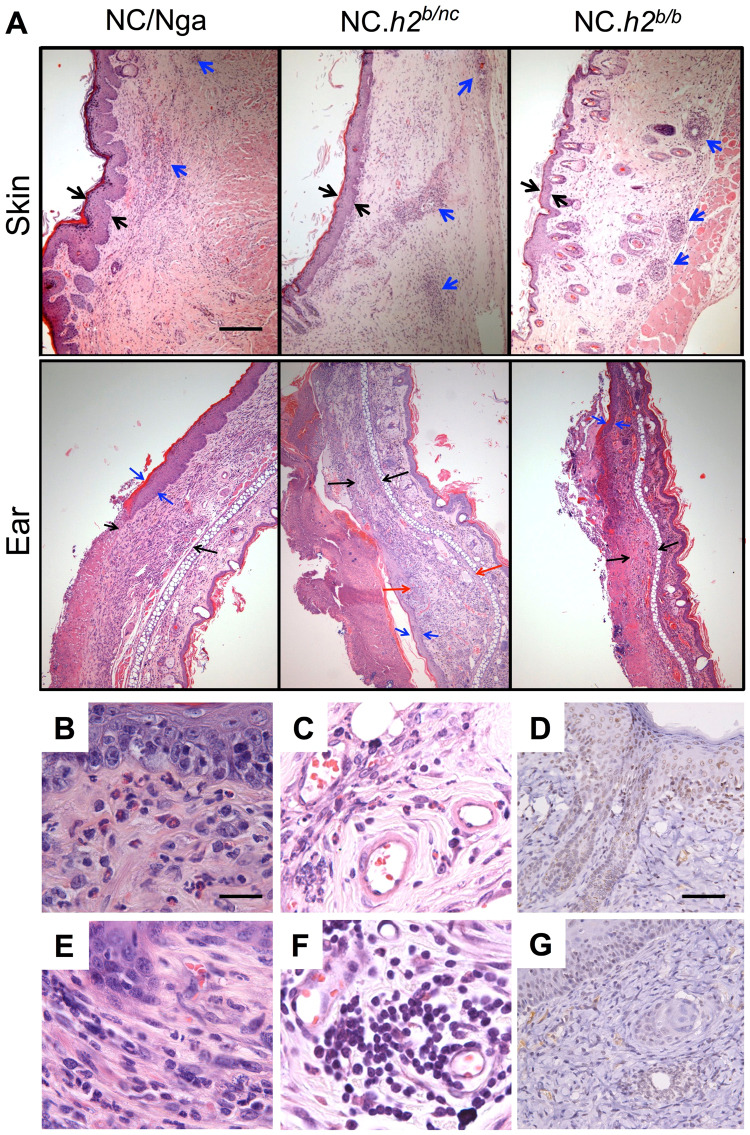
Histologically on DNFB-induced dermatitis, contact dermatitis was induced in NC.*h2*^*b/nc*^ and NC.*h2*^*b/b*^ while AD-like lesions were induced in NC/Nga. (**A**) Thickening of the epidermis (black arrow) and the perivasculitis of the dermis (blue arrow) in upper panel (skin) are indicated. Thickening of the epidermis (blue arrow) and dermis (black arrow) in lower panel (ear) are indicated. Partially extended thickening of the dermis is highlighted (red arrow). Scale bar corresponds to 100 µm in all images of (**A**). Against the infiltration of eosinophils in the dermis (**B**) and in perivascular (**C**), and the TSLP expression in epidermis (**D**) in NC/Nga, the infiltration of lymphocytes and plasma cells in the dermis (**E**) and in perivascular (**F**), and the TSLP expression in perivascular (**G**) in NC.*h2*^*b/b*^ is indicated. Scale bar in (**B**), (**C**), (**E**), (**F**) represents 25 µm. Scale bar in (**D**) and (**G**) is 50 µm.

Histologically, DNFB application induced dermatitis lesions in all mice including NC/Nga, NC.*h2*^*b/nc*^ and NC.*h2*^*b/b*^ (Fig. 3A). Thickening of the epidermis and dermis in the skin or ear were strongest developed in the following order: NC/Nga < NC.*h2*^*b/nc*^ < NC.*h2*^*b/b*^ (Fig. 3A). A sign of AD-like lesion accompanied by band-like infiltration of neutrophils, eosinophils and macrophages in the dermis was observed in NC/Nga (Fig. 3B,C). Additionally, signs of ACD-like lesions^20^ accompanied by fibroblast proliferation in dermis (Fig. 3E) and distinctive perivasculitis with infiltrating lymphocytes and plasma cells (Fig. 3F) were mainly observed in both NC.*h2*^*b/nc*^ and NC.*h2*^*b/b*^. Although high expression levels of thymic stromal lymphopoietin (TSLP) was remarkably detected in the NC/Nga epidermis (Fig. 3D), TSLP expression was observed in perivascular tissues rather than in the epidermis in NC.*h2*^*b/nc*^ and NC.*h2*^*b/b*^ (Fig. 3G). Although it was at a low level, serum TSLP levels also significantly increased in DNFB-induced dermatitis of NC/Nga than in that of NC.*h2*^*b/b*^ (p < 0.05, *ANOVA*; Supplemental Figure S1A,B). Furthermore, serum TNF-α and CCL2 levels in DNFB-applied NC/Nga also partially increased compared with those in DNFB-applied NC.*h2*^*b/b*^. However, there is no difference in serum IL-1β levels in both mice (Supplemental Figure S1A, B).

### **In axillary lymphadenopathy with DNFB-induced dermatitis, the production of IFN-γ and IL-17 from autoreactive CD4**^+^**T-cells in NC**.***h2***^***b/b***^**were enhanced compared with that in NC/Nga**

**Figure 4:**
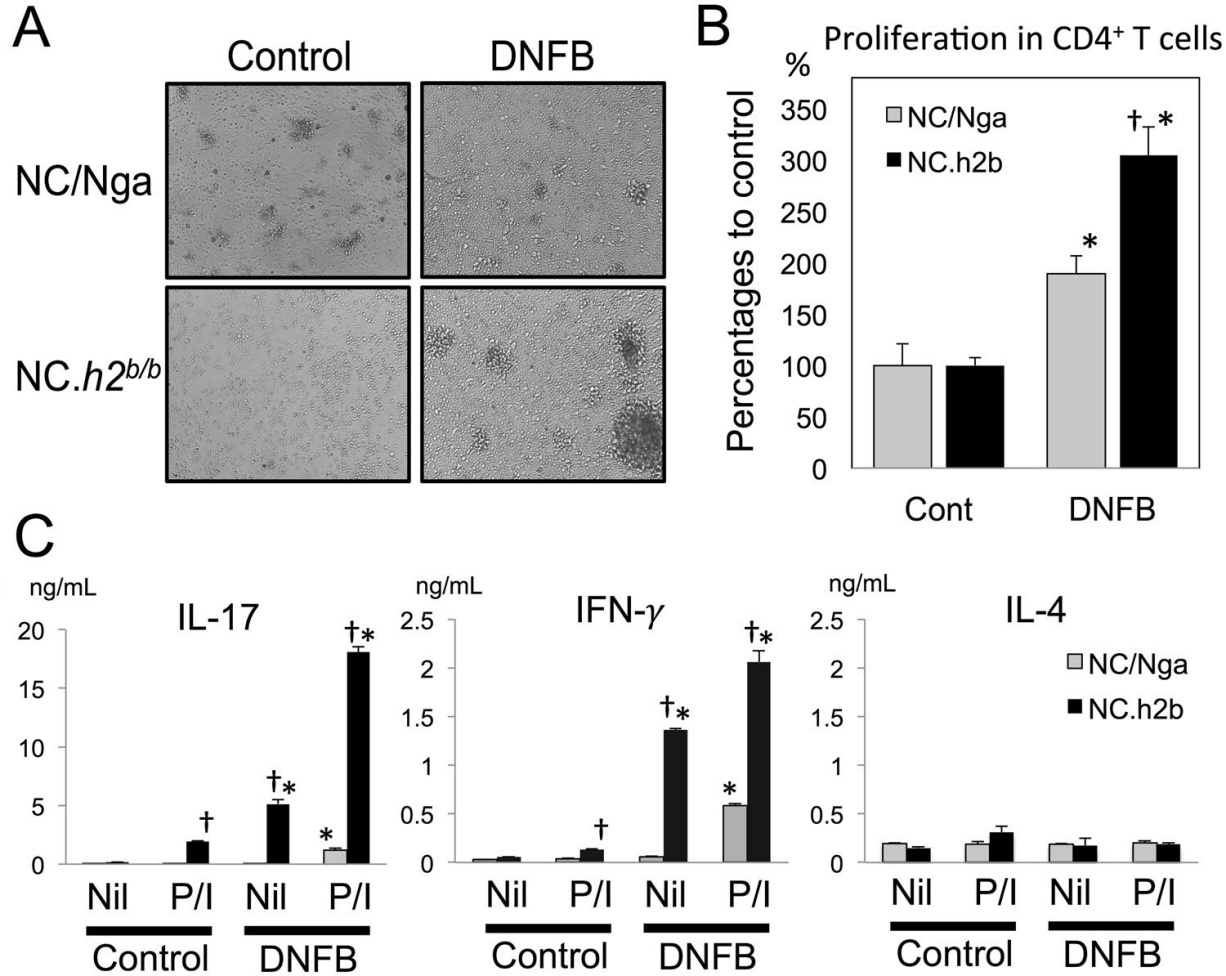
In axillary lymph nodes (LNs) with DNFB-induced dermatitis, the production of IFN-γ and IL-17 from autoreactive CD4^+^ T-cells in NC.*h2*^*b/b*^ is increased compared with that in NC/Nga. The autoreactive proliferation of CD4^+^ T-cells purified from axillary LNs was analyzed by co-culturing with naïve CD11b^+^ myeloid cells from the same strain. Growth picture (**A**) and graph of MTT assay (**B**) are indicated. *DNFB *vs*. control, p < 0.05, *ANOVA*. ^†^NC.*h2*^*b/b*^ *vs*. NC/Nga, p < 0.05, *ANOVA*. (**C**) The levels of IL-17, IFN-γ and IL-4 in culture supernatant was analyzed by the ELISA method. P/I; PMA^+^ Ionomycin. *DNFB *vs*. control, p < 0.05, *ANOVA*. ^†^P/I *vs*. Nil, *p* < 0.05, *ANOVA*. The bar graphs (n = 4) are indicated as mean ± SD.

Mice with DNFB-induced dermatitis exhibit an axillary lymphadenopathy phenotype (data not shown) that we further investigated in this study. As IL-4, IFN-γ and IL-17 are hardly detected in serum, we assessed their productions from CD4^+^ T-cells in axillary lymph nodes (LNs) by co-culture of these CD4^+^ T-cells with naïve CD11b^+^ myeloid cells (as antigen-presenting cells) whose growth response was stopped by X-ray irradiation. The productions of IFN-γ and IL-17, but not of IL-4, from proliferating autoreactive CD4^+^ T-cells in NC.*h2*^*b/b*^ were remarkably detected compared with NC/Nga (p < 0.05, *ANOVA*) (Fig. 4A–C). This result indicates that the total IgE production in DNFB-induced dermatitis of the NC/Nga strain is not dependent on IL-4-based mechanisms because IL-4 was hardly detected.

## Discussion

Here, we investigated the role of the *H2-*haplotype in the NC/Nga genomic background using *H2*-congenic mice (NC.*h2*^*b/b*^, NC.*h2*^*b/nc*^ and NC.*h2*^*nc/nc*^) established by backcrossing the NC/Nga strain. Severe dermatitis induced by repeated DNFB-application under SPF condition in NC/Nga was alleviated partially in NC.*h2*^*b/nc*^ and significantly in NC.*h2*^*b/b*^ as shown by decreased epidermal expression levels of TSLP and serum levels of total IgE, TSLP, IL-18 and IL-33. Histologically, ACD-like lesion of NC.*h2*^*b/nc*^ and NC.*h2*^*b/b*^ differed from AD-like lesions observed in NC/Nga. With the poor expression of IL4, the total IgE production in DNFB-induced dermatitis of NC/Nga is likely stimulated via an IL-4-independent mechanism.

The IL-1 family members IL-18 and IL-33 are highly inflammatory cytokines constitutively expressed in barrier cell types^21^, acting as regulators of innate and acquired immune responses by amplifying both Th1 and Th2 responses with or without TCR activation^22^. IL-18 and IL-33 signal their biologically activities through the heterodimeric receptors IL-18R and IL-33R in mast cells. IL-18 serum levels are elevated in AD patients or AD-induced NC/Nga mice^23,24^. IL-33 is an important mediator of allergy through its ability to induce Th2 cytokines and has been associated with development of severe AD^25^. IL-33 initiates allergic inflammation by activating innate lymphoid cells type 2 (ILC2s) to produce large amounts of Th2 cytokines IL-5 and IL-13 responsible for eosinophilia and IgE-class switching, respectively^7,26–28^.

Our results indicate that autoreactive CD4^+^ T-cells in axillary lymphadenopathy of DNFB-sensitised mice produce increased levels of IFN-γ and IL-17 in NC.*h2*^*b/b*^ compared with NC/Nga. Autoreactive CD4^+^ T-cells were previously reported to be induced in the DNFB-induced ACD model^29^. We confirm in this study that autoreactive CD4^+^ T-cells lead to ACD-like lesions in both DNFB-induced NC.*h2*^*b/b*^ and BALB/c mice. Although there are no reports on autoallergens specific to autoreactive CD4^+^ T-cells in ACD-developed mice, it is conceivable that autoreactive CD4^+^ T cells recognizing a carrier-protein of DNFB (a hapten) as self-antigens may be generated. In humans, the autoallergens Hom s 1–5 had been identified by screening of a human epithelial complementary DNA library for IgE binding of sera from AD patients^30–32^. Hom s 2 corresponds to the alpha-chain of the nascent polypeptide-associated complex (α-NAC) and α-NAC-specific autoreactive CD8^+^ T-cells were identified as secreting both IL-4 and IFN-γ in AD patients^33^. However, autoreactive CD4^+^ T-cells from PBMCs are not generated by α-NAC peptides in AD patients^34^. Recently, we detected IFN-γ-producing autoreactive CD8^+^ T-cells from NC/Nga mice with DNFB-induced AD that were expanded by co-culturing with dendritic cells treated with proteasome inhibitor MG132 (data not shown). Although an MHC class I mutant D/L^dm7^ has been downregulated by degradation with proteasome activation, the increase in D/L^dm7^ by MG132 addition was associated with the generation of autoreactive CD8^+^ T-cells, but not of autoreactive CD4^+^ T-cells (data not shown). Thus, autoreactive CD8^+^ T-cells or CD4^+^ T-cells are induced in AD-like lesions or in ACD-like lesions, respectively. Although it is a matter of speculation, the autoreactive Th1/Th17-like cells detected in ACD-like lesions may be inhibited *in vivo* by immunosuppressive cells other than CD4^+^ T-cells as these lesions display a weak inflammation phenotype compared with AD-like lesions and no sign of autoimmune disease and may play a role of inhibiting the generation of autoreactive CD8^+^ T-cells in changing AD-like lesions into ACD-like lesions.

## Materials and Methods

### Mice

NC/Nga (H-2^nc^) (male and female) and C57BL/6 (H-2^b^) mice (male) (Japan SLC, Hamamatsu, Japan) were maintained in SPF conditions, and used at 18–20 weeks of age. *H2*-congenic NC/Nga strain (NC.*h2*^*nc/nc*^, NC.*h2*^*b/nc*^ and NC.*h2*^*b/b*^) were generated by backcrossing (8 generations) of NC/Nga mice. Mice were maintained by breeding in SPF air conditions with microbiological monitoring tests (twice/year). All mice were maintained in our full-barrier animal facility under controlled temperature, humidity and 12 hour light/dark regimen. All experiments were approved by the Institutional Review Board for animal studies of NVLU, and performed following the guidelines provided by the Committee.

### Flowcytometry

Peripheral blood mononucleated cells (PBMCs) were isolated from mouse blood using Lymphoprep (ProGen, Heidelberg, Germany). For analysis, PBMCs suspended in PBS were stained with a mixture of FITC-conjugated K^d^ (SF1-1.1), I-A^b^ (KH74), I-A^k^ (11-5.2), I-E^k^ (14-4-45), D^b^ (27-11-13S)-specific mAbs (BioLegend, St. Louis, MO) and PE-conjugated K^b^ (AF6-88.5), L^d^ (28-14-8)-specific mAbs (BioLegend). Two-color or one-color analysis was conducted by FACS (FACSCalibur, Nippon Becton Dickinson, Japan).

### 2,4-dinitrofluorobenzene (DNFB)-induced dermatitis model

The methods in this study have been described previously^2^. AD-like skin lesions were induced by the repeated application of 25 µl of 0.15% DNFB (Wako Pure Chemicals, Tokyo, Japan) in acetone/olive oil (3:1) to the skin of the ears, calva, and neck on days 0, 3, 5, 7, 9, 11, and 13. The control mice were applied the acetone/olive oil (3:1) alone as the vehicle of DNFB. Dermatitis was evaluated by assigning an inflammation score^19^. Briefly, inflammation of the face, ears, and the anterior part of the body was scored as follows: 0, none; 1, mild; 2, moderate; and 3, severe. This scoring was based on the severity of erythema/hemorrhage (e/h), edema (ed), excoriation/erosion (e/e), and scaling/dryness (s/d), and total points were evaluated as the severity of dermatitis.

### ***In vivo*Imaging for MPO activity**

A chemiluminescent *in vivo* reagent for monitoring inflammation (XenoLight RediJect Inflammation probe, ParkinElmer) was administrated by intraperitoneal (*i.p*.) injection at 120 μL /mouse. MPO activity was analyzed by using IVIS *in vivo* imaging (IVIS Lumina II, ParkinElmer) at 10 minutes post *i.p*. injection of the probe under Pentobarbital anesthesia (Somnopentyl, Kyoritsu seiyaku).

### ELISA

Blood samples were collected from the orbital sinus in mice by using hematocrit tube, and serum samples were obtained by centrifugation. The total serum IgE was measured by a mouse IgE ELISA kit (Shibayagi Co., Ltd., Gunma, Japan). Serum IL-18 was measured by mouse *IL-18* ELISA kit (MBL). Serum IL-33 was measured by mouse *IL-33* Quantikine ELISA kit (R&D system). Culture supernatants were collected from each assay. The IFN-γ and IL-4 were measured by mouse *Th1*/*Th2* ELISA Ready-SET-Go! (eBioscience). The IL-17 was measured by mouse *IL-17* Quantikine ELISA kit (R&D system). All assays were following the manufacturer’s instructions.

### Histopathology and immunohistochemistry

Histopathological analyses were performed on a minimum of forth animals per experimental group. Tissues were immersion-fixed in 10% buffered formalin and processed by routine methods for paraffin sectioning. Paraffin sections, 5 μm thickness, were stained with hematoxylin and eosin (H&E), and examined by light microscopy.

In immunohistochemistry, rabbit polyclonal TSLP antibody (1:500, Abcam Inc., Cambridge, MA) was added and mixed with biotin goat anti-rabbit secondary antibody (1:1500, Dako A/S, Glostrup, Denmark), followed by peroxidase-conjugated streptavidin (Dako A/S). Finally, the reaction to antigen was visualized by addition of 3,3′-diaminobenzidine-tetrahydrochloride-dihydrate (DAB) and counterstained with hematoxylin.

### MTT assay

The culture medium was RPMI 1640 containing 10% heat-inactivated FCS, L-glutamine, non*-*essential amino acids, sodium pyruvate, 2-ME, and penicillin-streptomycin (Wako, Tokyo, Japan). CD4^+^ T-cells and CD11b^+^ myeloid cells (3 × 10^5^/well) were enriched from LNs by using anti-mouse CD4 or anti-mouse CD11b Magnetic Particles-DM (BD Biosciences). CD4^+^ T-cells were co-cultured for 72 h at 37 °C in 5% CO_2_ with CD11b + myeloid cells (3 × 10^5^/well) irradiated 30 Gy X-ray (CP160, Faxitron, Wheeling, IL). The cell proliferation was analyzed by MTT assay based on WST-8 [2-(2-methoxy-4-nitrophenyl)-3-(4-nitrophenyl) -5-(2,4-disulfophenyl)-2H-tetrazolium, monosodium salt] uptake (CCK-8; Dojindo Molecular Technologies, Kumamoto, Japan). 10 μl CCK-8 was added for 4 h prior to the end of culture, and absorbance was measured at 450 nm with a microplate reader (Bio-RAD Labs, Hercules, CA, USA). The average of auto-response was calculated as 100%.

### Statistical analysis

Statistical analysis was performed with *ANOVA* using Excel (Microsoft) and StatPlus (AnalystSoft, Alexandria, VA). A p-value < 0.05 was considered significant. *ANOVA* test was performed after normal distribution test (Shapiro-Wilk test).

## Electronic supplementary material

Supplementary Infomation

## References

[1] Matsuda H (1997). Development of atopic dermatitis-like skin lesion with IgE hyperproduction in NC/Nga mice. Int Immunol.

[2] Tomimori Y, Tanaka Y, Goto M, Fukuda Y (2005). Repeated topical challenge with chemical antigen elicits sustained dermatitis in NC/Nga mice in specific-pathogen-free condition. J Invest Dermatol.

[3] Miller, J. F., Vadas, M. A., Whitelaw, A., & Gamble, J. H-2 gene complex restricts transfer of delayed-type hypersensitivity in mice. *Proc Natl Acad Sci USA* **72**, 5095–5098. PMC388882 (1975) 10.1073/pnas.72.12.5095 PMC388882 1082137

[4] Sauder DN, Katz SI (1983). Strain variation in the induction of tolerance by epicutaneous application of trinitrochlorobenzene. J Invest Dermatol.

[5] Katayama I, Tanei R, Yokozeki H, Nishioka K, Dohi Y (1990). Induction of eczematous skin reaction in experimentally induced hyperplastic skin of Balb/C mice by monoclonal anti-DNP IgE antibody: possible implications for skin lesion formation in atopic dermatitis. Int Arch Allergy Appl Immunol..

[6] Kühn R, Rajewsky K, Müller W (1991). Generation and analysis of interleukin-4 deficient mice. Science..

[7] Punnonen J (1993). Interleukin 13 induces interleukin 4-independent IgG4 and IgE synthesis and CD23 expression by human B cells. Proc Natl Acad Sci USA.

[8] Emson CL, Bell SE, Jones A, Wisden W, McKenzie AN (1998). Interleukin (IL)-4-independent induction of immunoglobulin (Ig)E, and perturbation of T cell development in transgenic mice expressing IL-13. J Exp Med..

[9] Thyssen JP, McFadden JP, Kimber I (2014). Themultiple factors affecting the association between atopic dermatitis and contact sensitization. Allergy..

[10] Rundle CW, Bergman D, Goldenberg A, Jacob SE (2017). Contact dermatitis considerations in atopic dermatitis. Clin Dermatol..

[11] Heishi M (2003). Gene expression analysis of atopic dermatitis-like skin lesions induced in NC/Nga mice by mite antigen stimulation under specific pathogen-free conditions. Int Arch Allergy Immunol.

[12] Cabanillas B, Novak N (2016). Atopic dermatitis and filaggrin. Curr Opin Immunol..

[13] Otsuka A (2014). Possible new therapeutic strategy to regulate atopic dermatitis through upregulating filaggrin expression. J Allergy Clin Immunol..

[14] Ohkusu-Tsukada K, Tsukada T, Takahashi K (2017). Clonal deletion of T cell repertoires with specific T cell receptor Vβ chains by two endogenous superantigens in NC/Nga mice. Biosci Biotechnol Biochem..

[15] Habu Y (2001). The mechanism of a defective IFN-gamma response to bacterial toxins in an atopic dermatitis model, NC/Nga mice, and the therapeutic effect of IFN-gamma, IL-12, or IL-18 on dermatitis. J. Immunol..

[16] Hirota T (2012). Genome-wide association study identifies eight new susceptibility loci for atopic dermatitis in the Japanese population. Nat Genet..

[17] Weidinger S (2013). A genome-wide association study of atopic dermatitis identifies loci with overlapping effects on asthma and psoriasis. Hum Mol Genet..

[18] Ohkusu-Tsukada, K., Yamashita, T., Tsukada, T, & Takahashi, K. Low expression of a Ddm7/Ldm7-hybrid mutant (D/Ldm7) in the novel haplotype H-2nc identified in atopic dermatitis model NC/Nga mice. *Gene Immun*. *In press*10.1038/s41435-017-0003-y 29282355

[19] Leung DY (1990). Thymopentin therapy reduces the clinical severity of atopic dermatitis. J Allergy Clin Immunol.

[20] Rowe A, Farrell AM, Bunker CB (1997). Constitutive endothelial and inducible nitric oxide synthase in inflammatory dermatoses. Br J Dermatol..

[21] Guo L (2009). IL-1 family members and STAT activators induce cytokine production by Th2, Th17, and Th1 cells. Proc. Natl. Acad. Sci. USA.

[22] Smithgall MD (2008). IL-33 amplifies both Th1- and Th2-type responses through its activity on human basophils, allergen-reactive Th2 cells, iNKT and NK cells. Int. Immunol..

[23] Tanaka H (2001). IL-18 might reflect disease activity in mild and moderate asthma exacerbation. J. Allergy Clin. Immunol..

[24] Tanaka, T. *et al*. Interleukin-18 is elevated in the sera from patients with atopic dermatitis and from atopic dermatitis model mice, NC/Nga. Int. Arch. Allergy Immunol. **125**, 236–240. 53821 (2001) 10.1159/000053821 11490156

[25] Schmitz J (2005). IL-33, an interleukin-1-like cytokine that signals via the IL-1 receptor-related protein ST2 and induces T helper type 2-associated cytokines. Immunity.

[26] Yasuda K (2012). Contribution of IL-33-activated type II innate lymphoid cells to pulmonary eosinophilia in intestinal nematode-infected mice. Proc Natl Acad Sci USA.

[27] Lopez AF (1998). Recombinant human interleukin 5 is a selective activator of human eosinophil function. J Exp Med..

[28] McKenzie GJ (1998). Impaired development of Th2 cells in IL-13-deficient mice. Immunity..

[29] Fairchild RL, Moorhead JW (1998). Soluble factors in tolerance and contact sensitivity to DNFB in mice. VIII. Regulation of T suppressor cell function by autoreactive T helper cells. Cell Immunol..

[30] Valenta R (1998). Molecular characterization of an autoallergen, Hom s 1, identified by serum IgE from atopic dermatitis patients. J Invest Dermatol.

[31] Natter S (1998). Isolation of cDNA clones coding for IgE autoantigens with serum IgE from atopic dermatitis patients. FASEB J.

[32] Hradetzky S, Werfel T, Rösner LM (2015). Autoallergy in atopic dermatitis. Allergo J Int..

[33] Roesner LM (2016). α-NAC-Specific autoreactive CD8^+^ T cells in atopic dermatitis are of an effector memory type and secrete IL-4 and IFN-γ. J Immunol..

[34] Hradetzky S (2014). Cytokine effects induced by the human autoallergen α-NAC. J Invest Dermatol..

