# Supplementary material for: Signs of atopic dermatitis and contact dermatitis affected by distinct H2-haplotype in the NC/Nga genetic background
Source: Sci Rep. 2018 Feb 7;8:2586. doi: 10.1038/s41598-018-21049-x (PMC5803211; doi:10.1038/s41598-018-21049-x)
Supplement: Supplementary file 1 — Supplementary Infomation [file 41598_2018_21049_MOESM1_ESM.pdf]

Supplemental information

[Title]

Signs of atopic dermatitis and contact dermatitis affected by distinct *H2*-haplotype in the NC/Nga genetic background

[Short Title]

AD sensitivity by distinct *H2*-haplotype in the NC/Nga background

Kozo Ohkusu-Tsukada[1], Daiki Ito[1], Yuki Okuno[1], Teruyo Tsukada[2] and Kimimasa Takahashi[1]

[1] Department of Veterinary Pathology, Nippon Veterinary and Life-science University (NVLU).

[2] Radiation Biology Team, Nishina Center for Accelerator-based Science, RIKEN.

[Keyword] H-2^{nc}, *H2*-congenic NC/Nga mice, DNFB, atopic dermatitis (AD), allergic contact dermatitis (ACD)

Correspondence: Kozo Ohkusu-Tsukada, Ph.D., DVM. Department of Veterinary Pathology, Nippon Veterinary & Life-science University (NVLU). 1-7-1 Kyonan-cho, Musashino, Tokyo, Japan. E-mail: tkd-oks@nvlu.ac.jp

Supplementary Figure

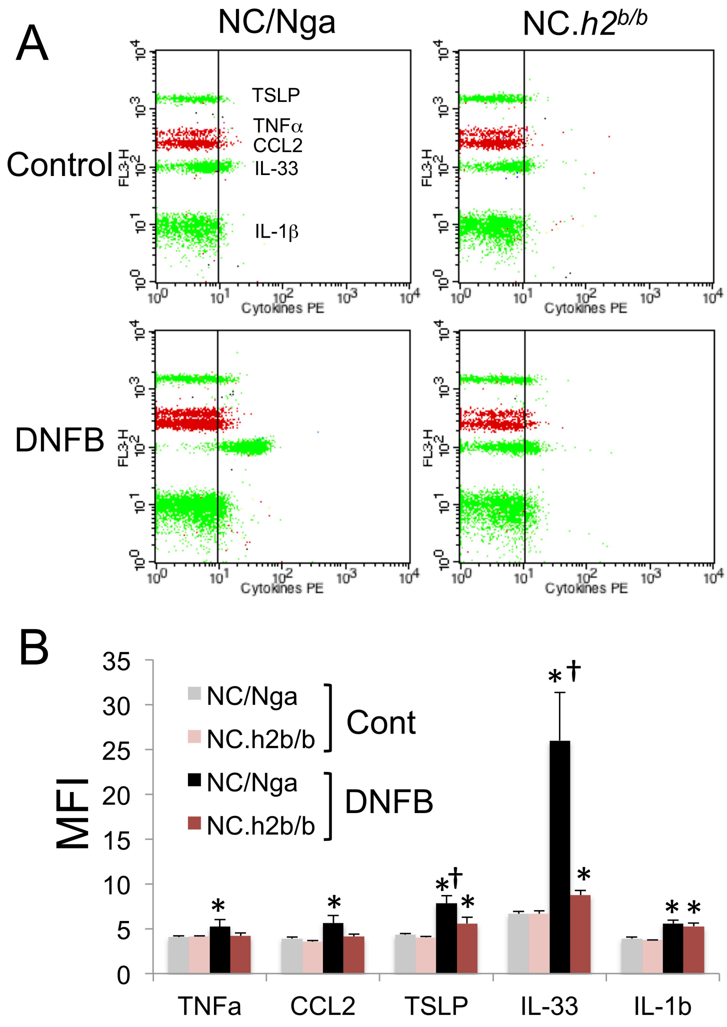

Supplemental Figure S1. The expression pattern of serum cytokines in NC/Nga and NC.*h2*$^{b/b}$ were analyzed by flow cytometry. **A.** A typical DATA of flow cytometry is shown. **B.** The bar graphs (n = 4) depicting the mean fluorescence intensity (MFI) are indicated as mean ± SD. * DNFB *vs.* control, $p < 0.05$, *ANOVA*. † NC/Nga *vs.* NC.*h2*$^{b/b}$, $p < 0.05$, *ANOVA*

Supplemental Material

Cytokines concentration in serum was determined using LEGENDplex™ Multi-Analyte Flow Assay Kit (BioLegend, TNFα-A7, CCL2-A8, IL-1β-B2, IL-33-B5, and TSLP-B9) according to the manufacturer's instructions.[1] Briefly, two size populations of bead A and bead B for the microsphere populations are distincted by FSC/SSC analysis. Each beads have a characteristic fluorescence intensity that can be detected with FL3, and were coated by each cytokine-specific mAbs. Serum cytokines are sandwiched with biotinylated each cytokine-specific mAbs on each mixed beads, followed to labeling with streptavidin-PE. The intensities in the classification fluorescence channels which are easy to gate and quantify are clearly defined by FL3/FL2 assays.

Reference

1. Lehmann J.S., *et al.* Multiplex cytokine profiling of stimulated mouse splenocytes using a cytometric bead-based immunoassay platform. *J Vis Exp.* 129: e56440. doi: 10.3791/56440 (2017)
